# 132例晚期非小细胞肺癌胸腔积液*EGFR*基因突变检测结果及其临床意义：一项来自单中心的回顾性研究

**DOI:** 10.3779/j.issn.1009-3419.2020.104.23

**Published:** 2020-12-20

**Authors:** 涛 鲁, 强 李, 岚 李, 凯珍 杨, 丹菲 周, 洁 高, 闽江 陈, 燕 徐, 巍 钟, 孟昭 王, 智勇 梁, 静 赵

**Affiliations:** 1 100730 北京，中国医学科学院北京协和医院病理科 Department of Pathology, Peking Union Medical College Hospital, Peking Union Medical College, Chinese Academy of Medical Sciences, Beijing 100730, China; 2 100007 北京，北京市第六医院呼吸内科 Department of Respiratory Medicine, Beijing No.6 Hospital, Beijing 100007, China; 3 050000 石家庄，石家庄市人民医院呼吸内科三病区 Department of Respiratory Medicine Ward 3, Shijiazhuang People's Hospital, Shijiazhuang 050000, China; 4 015000 巴彦淖尔，内蒙古巴彦淖尔市医院呼吸内科 Department of Respiratory Medicine, Bayannur City Hospital, Bayannur 015000, China; 5 315000 宁波，中国科学院大学宁波华美医院呼吸内科 Department of Respiratory Medicine, HwaMei Hospital, University of Chinese Academy of Sciences, Ningbo 315000, China; 6 100730 北京，中国医学科学院北京协和医院呼吸与危重症医学科 Department of Pulmonary and Critical Care Medicine, Peking Union Medical College Hospital, Peking Union Medical College, Chinese Academy of Medical Sciences, Beijing 100730, China

**Keywords:** 肺肿瘤, 胸腔积液, 表皮生长因子受体突变, 治疗, 病理质控, Lung neoplasms, Pleural effusionl, Epidermal growth factor receptor mutation, Treatment, Quality control standards

## Abstract

**背景与目的:**

恶性胸腔积液表皮生长因子受体（epidermal growth factor receptor, *EGFR*）基因突变检测缺乏病理质控，导致对检测结果解释及指导临床EGFR酪氨酸激酶抑制剂（tyrosine kinase inhibitor, TKI）使用造成困惑。因此，提出质控标准，并按此标准进行胸水*EGFR*突变检测尤为重要。本研究拟回顾性分析按照严格病理质控标准进行的胸水沉渣切片*EGFR*基因突变检测结果以及据此结果指导EGFR-TKIs治疗的疗效。

**方法:**

回顾性分析中国医学科学院北京协和医院病理科2012年1月-2018年6月收到的胸腔积液标本的患者临床资料，其中具有临床资料相对完整，且按照制定的质控标准进行了胸水沉渣石蜡包埋切片*EGFR*基因突变检测的患者132例。根据*EGFR*基因突变检测结果，分为阳性组和阴性组，比较不同组别使用EGFR-TKIs的疗效。

**结果:**

胸腔积液经离心后，沉渣石蜡包埋、切片，HE染色后镜下观察，若肿瘤细胞数目≥100个，即满足病理质控标准，可用于后续*EGFR*基因突变检测。132例患者中，72例（54.5%）患者胸水中检出*EGFR*基因突变。72例突变阳性患者中，69例患者使用了EGFR-TKIs。60例EGFR突变阴性患者中，仅15例使用EGFR-TKIs。*EGFR*突变阳性组的疾病控制率（disease control rate, DCR）为95.8%，中位无疾病进展生存时间（progression-free survival, PFS）为11个月；*EGFR*突变阴性组的DCR为0%，中位PFS为1个月，两组患者DCR和PFS均有显著差异（*P* < 0.05）。

**结论:**

经病理质控的胸水沉渣包埋切片可用于*EGFR*基因突变检测，其结果可指导临床EGFR-TKIs使用。

## Abstract (en)

**Background and objective:**

The lack of pathological quality control standard in detecting epidermal growth factor receptor (*EGFR*) gene mutation in malignant pleural effusion leads to confusion in the interpretation of detection results and the clinical use of EGFR-tyrosine kinase inhibitor (TKI). Therefore, it is very important to propose quality control standards and guide the detection of *EGFR* mutation in pleural effusion. The aim of this study is to retrospectively analyze the results of *EGFR* gene mutation in pleural effusion sediment section according to strict pathological quality control standards, and the therapeutic effect of EGFR-TKIs guided by this detection results.

**Methods:**

From January 2012 to June 2018, the clinical data of patients with pleural effusion collected from Department of Pathology of Peking Union Medical College Hospital were analyzed retrospectively. Among them, 132 patients with relatively complete clinical data and with *EGFR* gene mutation detection of paraffin-embedded pleural effusion sediment section according to the established quality control standard were included. According to the results of *EGFR* gene mutation, it was divided into positive group and negative group, and the efficacy of EGFR-TKIs in different groups was compared.

**Results:**

After the centrifugation of pleural effusion, the sediment was embedded in paraffin, sectioned, and observed under the microscope after HE staining. If the number of tumor cells ≥100, it met the pathological quality control standard, and it could be used for subsequent *EGFR* gene mutation detection. *EGFR* gene mutations were detected in 72 (54.5%) of 132 patients. EGFR-TKIs were used in 69 of 72 mutation positive patients. Of 60 *EGFR* mutation negative patients, only 15 used EGFR-TKIs. In *EGFR* mutation positive group, the disease control rate (DCR) was 95.8%, and the median progression-free survival (PFS) was 11 months. In *EGFR* mutation negative group, the DCR was 0%, and the median PFS was 1 month. The DCR and PFS were significantly different between the two groups (*P* < 0.05).

**Conclusion:**

According to the pathological quality control standards, the embedded section of pleural fluid sediment can be used to detect *EGFR* gene mutation, and the results can be used to guide the clinical use of EGFR-TKIs.

对于晚期非小细胞肺癌（non-small cell lung cancer, NSCLC），初始诊断时胸腔积液发生率为10%-15%；而对经治患者，于疾病后期出现胸膜转移或者胸腔积液的发生率更高^[1]^。由于恶性胸水中含有一定数量的肿瘤细胞，因此，使得胸水用于确诊以及进一步的分子检测成为可能。当前，胸腔积液表皮生长因子受体（epidermal growth factor receptor, *EGFR*）基因突变检测缺乏标准流程，大部分基因检测公司检测时缺乏严格的病理质控，造成胸水*EGFR*基因突变检测结果和组织检测结果有一定出入，对临床指导是否选择*EGFR*酪氨酸激酶抑制剂（EGFR-tyrosine kinase inhibitor, EGFR-TKI）进行治疗造成困惑。近期，一项纳入1, 226例东亚人群胸水*EGFR*突变检测的荟萃分析^[2]^显示，胸水标本*EGFR*突变检测的灵敏度仅为86%，分析原因可能与检测方法的敏感性以及缺乏病理质控有一定关系。

因此，本研究拟回顾性分析中国医学科学院北京协和医院病理科收集的胸腔积液标本的*EGFR*突变检测结果，重点阐述病理质控的标准操作流程以及该结果在真实世界中指导EGFR-TKIs使用情况及临床疗效。

## 1 资料和方法

### 1.1 纳入标准

①2012年1月-2018年6月期间，北京协和医院病理科分子病理室收到的胸腔积液标本的患者；②胸腔积液必须进行沉渣包埋及必要的免疫组化检查，用于诊断NSCLC；③胸水沉渣包埋切片用于病理质控，经判断满足标准，适合于进行*EGFR*基因突变检测；④所有患者均经组织病理学或胸水细胞学确诊为NSCLC；⑤病例资料相对完整，必须包括是否使用了EGFR-TKIs的信息。该回顾性研究经中国医学科学院北京协和医院伦理委员会批准进行，所有患者均签署知情同意书进行*EGFR*基因突变检测。

### 1.2 临床资料的收集

通过查阅病历，获取患者的临床基线特征及治疗信息。本研究收集以下数据：性别、年龄、吸烟史、病理类型、肿瘤原发灶-淋巴结-转移（tumor-node-metastasis, TNM）分期、EGFR-TKIs使用情况以及疗效，具体包括疾病控制率（disease control rate, DCR）和无进展生存时间（progression-free survival, PFS）。

### 1.3 疗效评价

依照实体瘤疗效评价标准（Response Evaluation in Solid Tumors, RECIST）1.1版，分为完全缓解（complete response, CR）、部分缓解（partial response, PR）、疾病稳定（stable disease, SD）和疾病进展（progressive disease, PD）。CR和PR必须在4周后经影像学检查确认。DCR指CR+PR+SD患者比率。PFS定义为开始EGFR-TKIs治疗至首次记录的PD时间或死亡时间。随访截止时间至2019年6月30日。

### 1.4 胸腔积液标本的处理方法

取胸水样本50 mL，3, 500 rpm，离心5 min后，弃上清，再加适量蛋清甘油凝固样本，并加入9倍于标本量的95%酒精混匀，3, 500 rpm，离心5 min后，沉渣石蜡包埋，备用。

### 1.5 能用于EGFR检测的胸腔积液标本质量控制标准

取上述石蜡包埋的沉渣，切片，HE染色。镜下观察，如果切片中，肿瘤细胞≥100个，即满足质控标准，可进行*EGFR*基因突变检测。如果切片中，肿瘤细胞 < 100个，则不满足质控标准，不推荐进行*EGFR*基因突变检测。如图 1所示。

**图 1:**
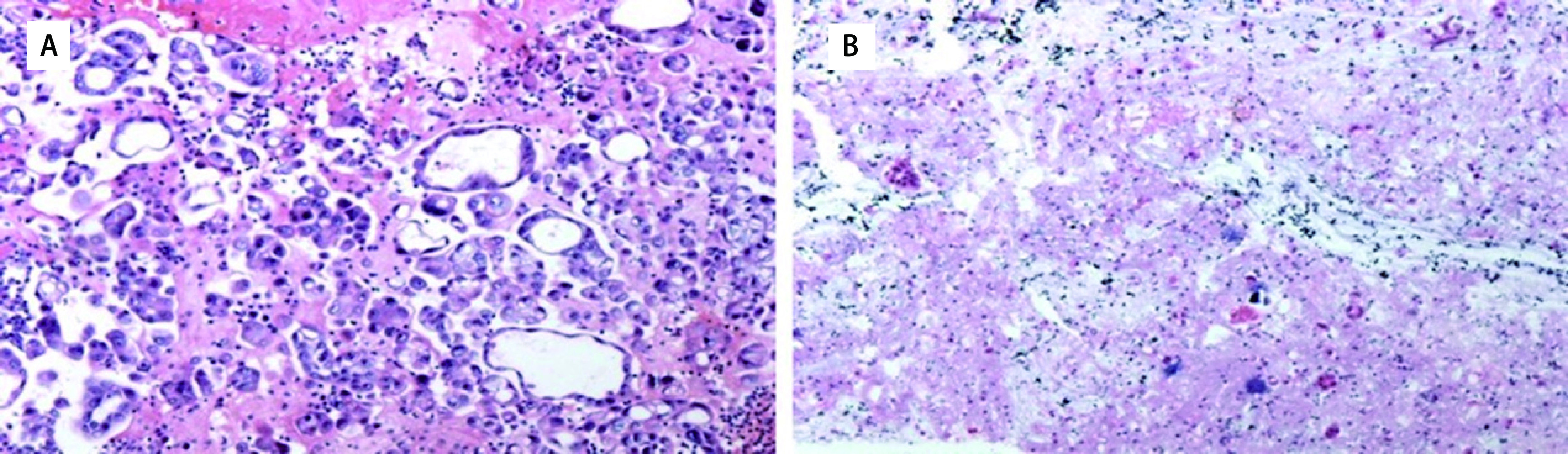
病理图片。A：胸水沉渣包埋切片提示有大量肿瘤细胞，部分排列呈腺管状及微乳头状，全片肿瘤细胞数目大于100，病理质控合格；B：胸水沉渣包埋切片提示肿瘤细胞少，肿瘤细胞单个散在或呈腺管状，全片肿瘤细胞数目小于100，病理质控不合格（HE染色, ×10）。 Pathological pictures. A: The section of pleural fluid sediment embedded in paraffin indicates that there are a large number of tumor cells, which are presented in tubular and papillary morphology. The number of tumor cells in the whole section is more than 100, and the pathological quality control is qualified; B: The section of pleural fluid sediment embedded in paraffin indicates that there are few tumor cells, which are scattered or presented in tubular morphology. The number of tumor cells in the whole section is less than 100, and the pathological quality control is unqualified (HE staining, ×10).

### 1.6 胸腔积液*EGFR*基因突变检测

满足上述质量控制标准的胸水沉渣蜡块，常规切片5 μm厚，5张-10张，刮片富集肿瘤细胞，提取DNA并定量。*EGFR*基因突变，采用市售*EGFR*基因突变检测试剂盒（德国凯杰EGFR RGQ PCR Kit，QIAGEN，批号：3540192，货号：870111），检测方法和步骤按照试剂盒操作说明书进行。

### 1.7 统计学方法

采用SPSS 21.0统计学软件进行数据统计学处理。计量资料采用中位数描述，计数资料采用率表示。率的比较采用卡方检验或秩和检验。均数的比较采用*t*检验。生存数据采用*Kaplan-Meier*分析并进行*Log-rank*检验。所以统计检验均为双侧检验，*P* < 0.05表示差异有统计学意义。

## 2 结果

### 2.1 本研究流程图

2012年1月-2018年6月，中国医学科学院北京协和医院病理科分子病理室收到的胸腔积液标本，按照胸水沉渣包埋切片中肿瘤细胞数目≥100个作为质控标准，共有155例胸腔积液标本进行了*EGFR*突变检测，排除资料不全、未确诊以及非NSCLC患者，最终132例患者纳入本研究。132例患者中，72例（54.5%）患者*EGFR*突变阳性，另60例（45.5%）患者*EGFR*突变阴性。72例突变阳性患者中，除2例20 Ins以及1例T790M突变患者未使用EGFR-TKIs，其余69例患者均使用了EGFR-TKIs。60例*EGFR*突变阴性患者中，有15例使用EGFR-TKIs。如图 2所示。

**图 2:**
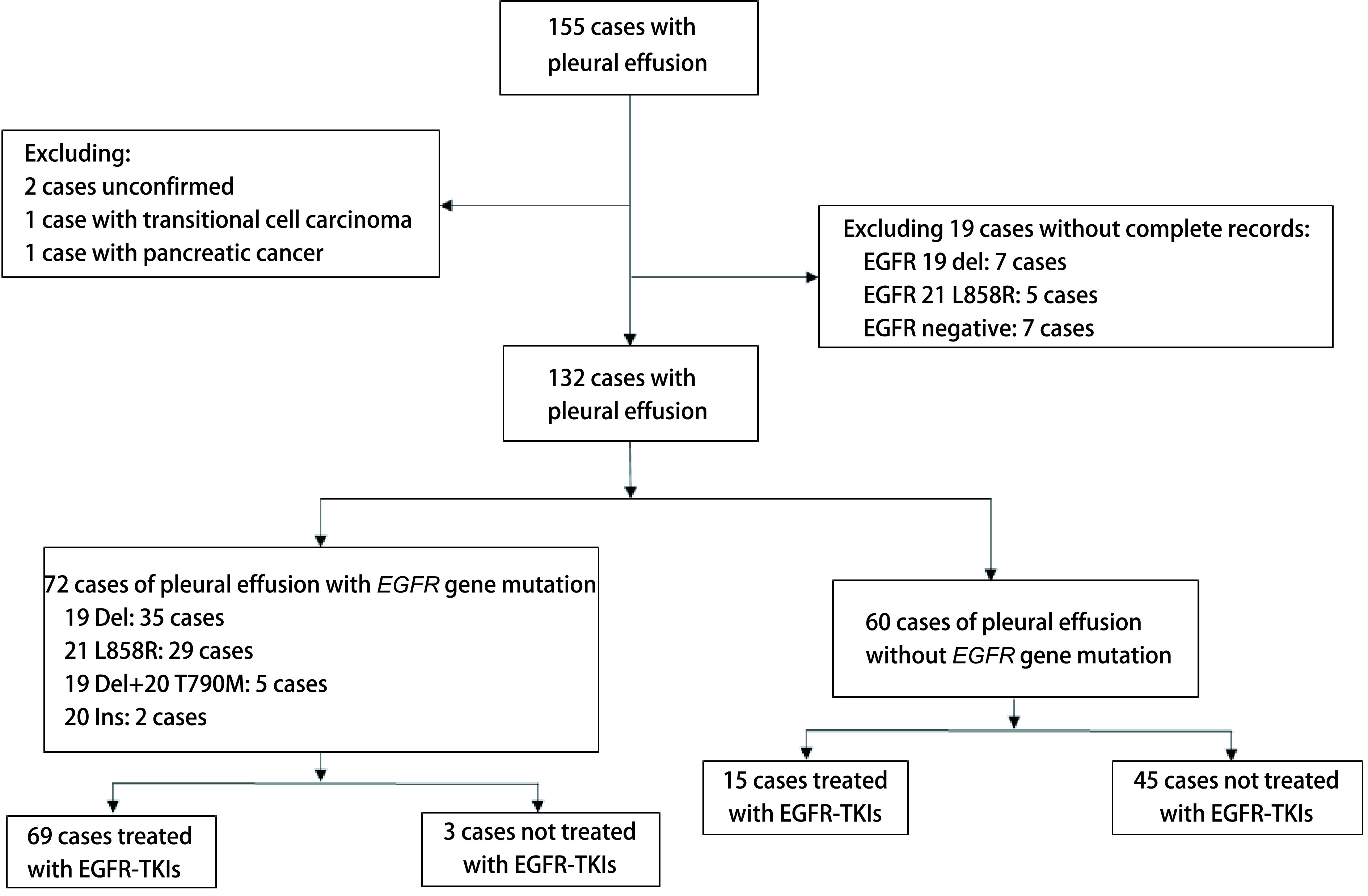
本研究流程图 The flow chart of this study

### 2.2 入组患者临床特征

132例患者中，男性64例（48.5%），女性68例（51.5%）；中位年龄63岁（54岁-74岁）；正在吸烟或既往有吸烟史者35例（26.5%），从不吸烟者97例（73.5%）；除1例（0.8%）为大细胞癌外，其余均为腺癌（131例，99.2%）；分期上均属于IV期。胸水*EGFR*基因突变阳性组和*EGFR*基因突变阴性组，在性别和吸烟方面并未显示存在显著差异，*P*值分别为0.382和0.24，如表 1所示。

**表 1:** 132例患者的临床资料[*n* (%)] Clinical characteristics of the 132 study patients [*n* (%)]

Characteristics	All (*n*=132)	*EGFR* positive (*n*=72)	*EGFR* negative (*n*=60)	*P*
Gender				0.382
Male	64 (48.5)	32 (44.4)	32 (53.3)	
Female	68 (51.5)	40 (55.6)	28 (46.7)	
Age [median (IQR)]	63 (54-74)	58 (41-75)	59.5 (43-70)	
Smoking status				0.240
Current/Former	35 (26.5)	16 (22.2)	19 (31.7)	
Never	97 (73.5)	56 (77.8)	41 (68.3)	
Histology				> 0.999
Adenocarcinoma	131 (99.2)	71 (98.6)	60 (100.0)	
Large cell	1 (0.8)	1 (1.4)	0 (0.0)	
EGFR-TKIs				< 0.001
Gefitinib	32 (24.2)	29 (40.3)	3 (5.0)	
Erlotinib	12 (9.1)	9 (12.5)	3 (5.0)	
Icotinib	38 (28.8)	29 (40.3)	9 (15.0)	
Osimertinib	2 (1.5)	2 (2.8)	0 (0.0)	
Not used	48 (36.4)	3 (4.2)	45 (75.0)	
Response to EGFR-TKIs				0.001
PR+SD	23 (85.2)	23 (95.8)	0 (0.0)	
PD	4 (14.8)	1 (4.2)	3 (100.0)	
PFS during EGFR-TKIs treatment (mon, median, 95%CI)	10.3 (8.9, 11.8)	11.0 (8.9, 13.1)	1.0 (0.7, 1.3)	< 0.001
PFS: progression-free survival; PR: partial response; SD: stable disease; PD: progressive disease; EGFR-TKIs: epidermal growth factor receptor tyrosine kinase inhibitor.				

### 2.3 入组患者*EGFR*基因突变情况分布

72例*EGFR*基因突变中，19 Del和21 L858R是主要突变类型，分别为35例和29例，占*EGFR*基因突变的88.9%；原发耐药突变20 T790M 1例（1.4%）以及20 Ins 2例（2.8%）；另外，*EGFR*基因双突变5例（6.9%），且均为19 Del合并20 T790M。

### 2.4 132例患者EGFR-TKIs使用情况

132例患者中，84例（63.6%）患者使用了EGFR-TKIs，其中32例（38.1%）使用吉非替尼，12例（14.3%）使用厄洛替尼，38例（45.2%）使用埃克替尼，2例（2.4%）使用奥希替尼。在72例*EGFR*突变阳性患者中，69例（95.8%）患者使用了EGFR-TKIs，其中29例（40.3%）选择吉非替尼，9例（12.5%）选择厄洛替尼，29例（40.3%）选择埃克替尼，3例（4.2%）未选择EGFR-TKIs治疗（1例为20 T790M突变，2例为20 Ins）；在这69例患者中，将EGFR-TKIs作为一线治疗59例（85.5%），作为二线治疗10例（14.5%）。在60例*EGFR*突变阴性患者中，15例（25%）患者使用了EGFR-TKIs，其中3例（5%）选择吉非替尼，3例（5%）选择厄洛替尼，9例（15%）选择埃克替尼，45例（75%）患者未使用EGFR-TKIs；在这15例患者中，其中将EGFR-TKIs作为一线治疗6例（40%），二线治疗4例（26.7%），三线及以上5例（33.3%）。

### 2.5 EGFR-TKIs治疗的DCR

*EGFR*突变阳性组中24例患者有疗效评估结果，无CR出现，23例出现PR+SD，1例出现PD，DCR为95.8%。*EGFR*突变阴性组中3例患者有疗效评估结果，且均为PD，DCR为0%。*EGFR*突变阳性组和突变阴性组中使用EGFR-TKIs在疗效上有显著差异（*P*=0.001）。

### 2.6 EGFR-TKIs治疗的PFS

至末次随访时，*EGFR*突变阳性组中69例使用EGFR-TKIs的患者中，43例出现PD，中位PFS为11个月（95%CI: 8.89-13.11）；*EGFR*突变阴性组中15例使用EGFR-TKIs的患者中，6例出现进展，中位PFS为1个月（95%CI: 0.70-1.30）。两组采用*Log-rank*检验，*P* < 0.001，有显著差异。两组PFS曲线如图 3所示。

**图 3:**
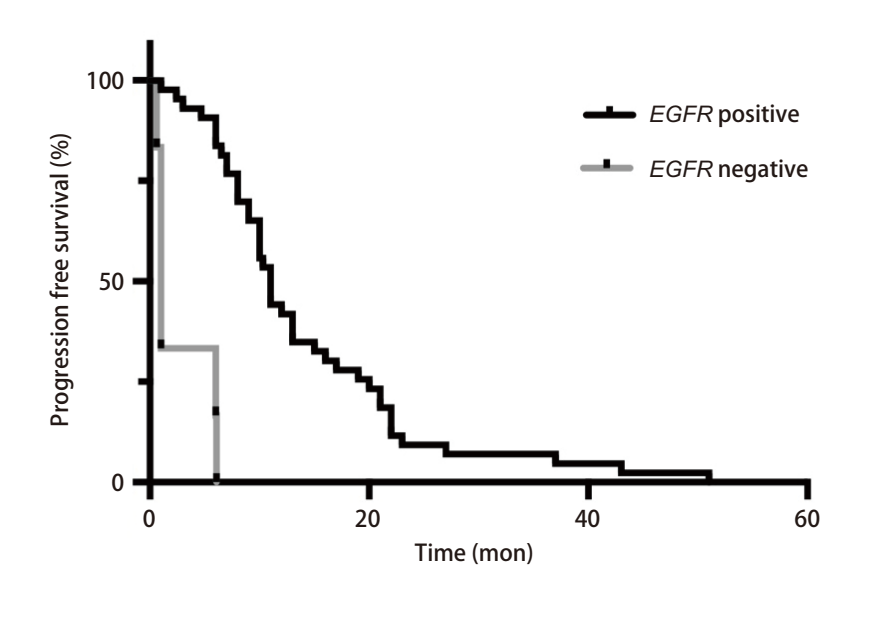
胸水*EGFR*突变阳性和阴性患者使用EGFR-TKIs的PFS曲线 PFS for *EGFR* positive and negative paitents treated with EGFR-TKIs

## 3 讨论

目前，关于胸水检测*EGFR*基因突变的研究主要集中在方法学上，包括传统测序^[3]^、下一代测序技术（next generation sequencing, NGS）^[4, 5]^、肽核酸PCR（peptide nucleic acid-PCR, PNA-PCR）测序^[3, 6]^、扩增阻滞突变系统（amplification refractory mutation system, ARMS）^[7]^、微滴式数字PCR（droplet digital PCR, ddPCR）^[8]^、cobas^[9]^等。一般来说，高灵敏度方法，如cobas、ddPCR或ARMS法较传统测序以及NGS等方法具有更高的敏感性。另外，有数项研究比较胸水离心后的上清液和胸水沉渣包埋细胞块用于检测*EGFR*基因的敏感性，结果有争议，部分研究^[4, 7]^显示两者检测结果一致性较高，而另外有研究^[10]^显示胸水沉渣细胞块的敏感性高于胸水上清液。一篇纳入15项东亚人群胸水*EGFR*突变检测研究的*meta*分析^[2]^显示，相对于肿瘤组织，胸水标本（包括胸水上清液或胸水沉渣包埋细胞块）*EGFR*检测敏感性为86%，特异性为93%，存在14%的漏诊率以及7%的误诊率，究其原因可能与胸水标本未进行严格病理质控有关。同时这也提示，基因检测首选肿瘤组织；对于无法获取组织的晚期NSCLC患者，可用胸水标本替代组织进行*EGFR*基因突变检测。

关于胸水*EGFR*基因检测的病理质控研究很少，很少有研究关注如何做好胸水检测*EGFR*基因突变的质控问题。有学者^[7, 10]^认为，胸水*EGFR*基因突变检测的质控取决于所选用的方法，如ARMS法的检测灵敏度为1%，因此，要求胸水沉渣切片中肿瘤细胞比例须大于1%-2%。一般而言，胸水中肿瘤细胞含量越高或者富集胸水中肿瘤细胞，胸水沉渣EGFR检测敏感性越高^[5, 11]^。2016版中国NSCLC患者*EGFR*基因突变检测专家共识^[12]^明确指出，无论采用哪种标本类型，均应保证包含足够的肿瘤细胞，尽量剔除非肿瘤组织和细胞，推荐肿瘤细胞数量在200个以上，肿瘤细胞比例达50%，应用灵敏度高的方法时可酌情降低标准。由于采用了敏感的ARMS检测方法，中国医学科学院北京协和医院分子病理科分子病理室将胸水沉渣细胞块的质控标准规定为肿瘤细胞≥100个，并以此标准指导临床样本检测。

本回顾性研究显示，胸水沉渣细胞块*EGFR*基因突变阳性率为54.5%，而且主要是*EGFR*经典突变19 Del和21 L858R，此与文献^[3, 4, 7-10]^中报道的结果一致。既往研究^[13-15]^显示，*EGFR*基因突变更常见于女性及不吸烟患者。但也有研究^[16]^显示，对于有胸腔积液的肺腺癌患者，*EGFR*基因突变与性别、吸烟史、年龄及肿瘤分期均无关，此与本研究结果一致。另外，本研究未能提示的这些特征，也可能与样本量少及严格病理质控后导致更大选择性偏倚有关，但严格的病理质控是为了保证检测结果的准确、可靠。本研究中，*EGFR*突变阳性组，使用EGFR-TKIs，DCR高达95.8%，中位PFS为11个月，而*EGFR*突变阴性组，使用EGFR-TKIs均在1个月后评估病情，出现PD，中位PFS仅1个月，疗效指标均具有显著差异，这与其他研究结果^[3, 4, 6]^一致。虽然胸水沉渣*EGFR*基因突变检测结果，无配对肿瘤组织检测结果进行验证，但是该检测结果指导下的EGFR-TKIs使用及其疗效与既往通过组织检测结果指导EGFR-TKIs使用疗效一致^[17-19]^，这也间接说明了具有病理质控把关前提下的胸水沉渣*EGFR*突变检测结果准确可靠，具有较强的临床意义。

本次回顾性研究的132例患者中，84例（63.6%）患者选择了EGFR-TKIs作为一种治疗手段，其中32例（38.1%）使用吉非替尼，12例（14.3%）使用厄洛替尼，38例（45.2%）使用埃克替尼，仅2例（2.4%）使用奥希替尼。由于一代EGFR-TKIs之间疗效差异不明显，但副作用谱还是有明显区别的。其中，厄洛替尼的皮疹、腹泻发生率明显高于吉非替尼、埃克替尼^[20]^。此外，二代EGFR-TKIs较一代EGFR-TKIs，皮疹、甲沟炎以及腹泻发生率显著升高^[21, 22]^。因此，在临床治疗抉择中，一代吉非替尼和埃克替尼的使用率远远高于厄洛替尼。尽管三代奥希替尼与一代吉非替尼相比，能够显著提高PFS和总生存期（overall survival, OS）^[23]^，不良反应也不明显，但因经济原因，一线选择奥希替尼治疗也非常少，本研究中仅2例使用奥希替尼，其中1例是同时合并19 Del和20 T790M突变，另1例为19 Del突变。

多项研究^[17-19, 21-23]^证实，对于*EGFR*突变阳性患者，EGFR-TKIs较化疗能够明显延长PFS，同时不良反应大大下降，因此，众指南均将EGFR-TKIs推荐为一线治疗。在本研究中，胸水*EGFR*突变阳性患者中，85.5%将EGFR-TKIs作为一线治疗，这也与指南推荐一致。

本研究也有很多局限性。第一，病理质控标准的确定属于经验性，应该进一步探讨不同质控标准下的检测敏感性及特异性，以及不满足质控标准的标本是否可以通过采用富集肿瘤细胞的方法，如显微切割等，提高肿瘤细胞比例，来提高检测敏感性，值得进一步研究；第二，缺乏配对的组织标本的检测结果，无法客观判断检测结果的准确性，另外没有同时评价胸水上清液是否适合作为组织检测的替代样本；第三，本研究数据失访、删失比例高，无法获得OS、客观缓解率（objective response rate, ORR）等数据用于统计分析；第四，本研究属于回顾性研究，而且样本量不大，所得结论尚需大规模前瞻性研究去进一步验证。

综上，采用胸水沉渣包埋细胞块切片中肿瘤细胞计数≥100作为质控标准，指导进一步进行*EGFR*基因突变检测所得结果能够指导临床选择EGFR-TKIs进行治疗，具有较强的临床意义，但其中尚有更多细节，比如质控标准的优化，更适宜推广的检测方法，胸水上清液检测的评价等尚需要进一步研究。
